# Prolonged Constipation

**Published:** 1888-01

**Authors:** W. S. Gee

**Affiliations:** Hyde Park, Ill.


### PROLONGED CONSTIPATION.

DR. W. S. GEE, HYDE PARK, ILL.

The case reported by Dr. Elliott in November issue is remarkable in many particulars. One of the most remarkable features was the persistence of the Doctor in letting good enough alone. He evidently "knows a thing or two" of the human system and of Homœopathy. I wish to record a case now under treatment. Mrs. B., æt. fifty, apparently well for her, of nervous temperament, has had a lifetime of constipation. Her brother-in-law is a prominent allopathic surgeon in Chicago, and has racked his brain and bothered his colleagues not a little to relieve this lady.

The whole list of purgatives have passed through, some singly, with a slow plod, and others in combinations innumerable, have gone with a "hop-step-and-jump," sweeping everything before them, and not stopping for two or three days afterward, but the old trouble returned; so, for years she did nothing.

The "enemas" or "clysters," in reality *rectal injections*, were used on special occasions. It was her habit to go two or three weeks without movement, and of late years she very frequently goes *six weeks* with no inclination to movement. This interval has been common for several years. Then she empties out in two or three days and dispenses with that function for another such interval.

I have given her an occasional prescription for some new trouble, and perhaps one call would suffice for the time. She has been abroad for months at a time. At my request she called on Dr. Skinner, but twice found him out. She called on a physician in Germany who gave her one powder, with a request to call again "in four weeks," but she received no effect and did not see him again.

On inquiry she stated that during the interval of bowel suspense the urine was much increased. The stools are not peculiar. About two weeks since I gave her a dose of Alumen$^{1m}$ (Skinner), but have had no report since.

In Vol. IV, p. 238, of *The Clinique*, Dr. Bailey has detailed a remarkable case in which Morphia hypodermically seemed to be the only agent used which produced a movement of the bowels. The intervals were: "February 13th to April 28th, unchanged, no movement of the bowels whatever; May 1st to 28th; May 30th to June 17th; June 30th to July 13th."

He there states that the constipation appeared soon after the disappearance of an abscess in the lower portion of left lung, (probably.)

Phthisis was hereditary.

In Vol. V, p. 449, of same journal, he gave a subsequent report of the case with *post-mortem* record.

"After an illness of more than five years my patient died, October 31st, 1884, of phthisis pulmonalis. * * * The bowels were inactive, the intervals varying from one to nine weeks."

Atrophic changes were marked in various organs, "the peritoneum was as thin as tissue paper and glued by adhesions to the intestines. * * * The stomach was divided by constricting peritoneal bands, so as to separate it into two quite distinct compartments of nearly equal size, and as an organ was enlarged and distended. * * * The omentum was firmly adherent, producing a serious knotting together of the entire small intestines. The transverse colon was greatly distended and enlarged, and pouched by constrictions the same as already noted. At the splenic flexure there was a great band, which by encircling the colon had caused an abrupt narrowing of its calibre, as great as the change in diameter of a wrist to that of an index finger, which was literally true in this case. This narrowing of the descending colon was continued until it had reached the signoid flexure; here a congenital malformation was noted, the colon traversing across the lower abdomen, a curve being found at a point just over the ilio-cœcal valve, from thence passing downward and backward terminated in an enormous pouch which completely filled the inferior outlet of the pelvis. All shape of the rectum had been lost. * * * The bladder was shrunken to the size of a walnut. * * * The constipation was a result of pathological changes, and in part congenital. The disease was tubercular."

My patient does not show a tubercular tendency, but may later. I think the above will give us hints in the study of such cases.

